# Mitochondrial-Derived Reactive Oxygen Species Play a Vital Role in the Salicylic Acid Signaling Pathway in *Arabidopsis thaliana*

**DOI:** 10.1371/journal.pone.0119853

**Published:** 2015-03-26

**Authors:** Shengjun Nie, Haiyun Yue, Jun Zhou, Da Xing

**Affiliations:** MOE Key Laboratory of Laser Life Science & Institute of Laser Life Science, College of Biophotonics, South China Normal University, Guangzhou, China; Indiana University, UNITED STATES

## Abstract

Plant mitochondria constitute a major source of ROS and are proposed to act as signaling organelles in the orchestration of defense response. At present, the signals generated and then integrated by mitochondria are still limited. Here, fluorescence techniques were used to monitor the events of mitochondria *in vivo*, as well as the induction of mitochondrial signaling by a natural defensive signal chemical salicylic acid (SA). An inhibition of respiration was observed in isolated mitochondria subjected to SA. The cytochrome reductase activity analysis in isolated mitochondria demonstrated that SA might act directly on the complex III in the respiration chain by inhibiting the activity. With this alteration, a quick burst of mitochondrial ROS (mtROS) was stimulated. SA-induced mtROS caused mitochondrial morphology transition in leaf tissue or protoplasts expressing mitochondria-GFP (43C5) and depolarization of membrane potential. However, the application of AsA, an H2O2 scavenger, significantly prevented both events, indicating that both of them are attributable to ROS accumulation. In parallel, SA-induced mtROS up-regulated *AOX1a* transcript abundance and this induction was correlated with the disease resistance, whereas AsA-pretreatment interdicted this effect. It is concluded that mitochondria play an essential role in the signaling pathway of SA-induced ROS generation, which possibly provided new insight into the SA-mediated biological processes, including plant defense response.

## Introduction

Studies during the last two decades have established that plant hormone salicylic acid (SA) is implicated in plant defense response to pathogen attack [1–3]. SA levels dramatically increase in the infected plant cells often following a hypersensitive response (HR), a form of resistance that is characterized by localized cell death at or around the initial point of pathogen entry [4]. In addition to this local accumulation, SA is required for the induction of systemic acquired resistance (SAR) against a wide variety of pathogens [5–7]. Besides its effects in response to biotic stress, SA plays an important role in plant responses to abiotic stress, particularly ozone [8], heat stress [9, 10], and salt and osmotic stresses [11, 12].

The production of reactive oxygen species (ROS) is a hallmark in the recognition of pathogens. A feature of ROS signaling is its interaction with plant hormone SA. For example, at the site of pathogen penetration, SA accumulates and necrotic damage occurs, which is accompanied by production of ROS [13–16]. SA can also down-regulate ROS-scavenging systems that, in turn, contribute to increase overall ROS levels following pathogen recognition [17, 18]. During germination, *NahG* plants, which express a salicylate hydroxylase gene, germinated better than the wild-type under salt and osmotic stresses and remained green to develop true leaves, suggesting that SA also potentiates the generation of ROS during this stress [11]. Based on these data, it is important to uncover the mechanism of SA action in ROS generation.

Plant mitochondria have been proposed as target sites for the action of signaling molecules generated during plant-pathogen interactions and cellular metabolism [19–23]. Complex I and III of the electron transport chain (ETC) are recognized as the major sites for ROS production [16, 23, 24–26]. The mitochondrial ROS (mtROS) are generated through electron leaks from the electron transport system, depending on inhibition of specific sites in the ETC or the reduction state of the ETC components, as substrates are metabolized [27]. A well-characterized role of a complex III inhibitor, antimycin A (AA), is restricting electron flow and leading to an over-reduction of ETC components and accumulation of mtROS [28, 29]. During HR, mitochondria are proposed to be a death integrator; mtROS influence the behavior of the whole cell [7, 20, 30, 31]. After pathogen perception, mitochondria function in the defense strategy of the plant cell, integrating and amplifying diverse signals such as SA, ROS or pathogen elicitors. The signals perceived by mitochondria usually impact on their normal function, destabilizing the organelle, generating changes in respiration, ROS production and membrane potential, thus establishing defense mechanisms and modulating the immune response [7, 23, 32–34]. The natural defensive signal chemical SA could impact on mitochondrial function in a dose dependent manner, by inhibiting electron flow and altering respiration rate [35, 36]. In addition, either abiotic or biotic stresses raise ROS levels possibly due to perturbations of mitochondrial metabolism [37, 38]. Collectively, these findings indicate the importance of mitochondrial signal in support of plant stress responses. Despite extensive research on the source of ROS and biochemical properties of ROS in plants, the mechanism of mtROS production in response to SA and the characterization and role of mitochondria during this process have yet to be determined in detail.

The cyanide-insensitive alternative oxidase (AOX) is thought to play a potentially crucial role in the maintenance of plant homeostasis [39–41]. AOX catalyzes the oxidation of ubiquinol and reduction of oxygen to water, effectively acting as the unique respiratory terminal oxidase whereby electron flow bypasses complex III and IV [29]. Once the electron transport in the cytochrome c pathway is blocked, AOX helps to maintain the electron flux and to reduce mtROS levels [41–45]. In the AOX family, AOX1a is an important member and often dramatically induced at the transcript level by a variety of stresses [44], while other gene family members display tissue or developmental specificity in their expression [46]. Ordog *et al*. [47], Cvetkovska & Vanleberghe [31, 32], and Vanlerberghe *et al*. [48] found that AOX could be involved in HR and may prevent programmed cell death (PCD). AOX activity and transcription of *AOX* mRNA can be stimulated by the chemical inhibition of the cytochrome pathway, cyanide and AA, as well as by SA [49, 50]. Thus, the effective function of mitochondria in stress response indicates flexibility in plant, and such flexibility is considered as an essential mechanism which makes plants adapt better to stress.

This study was to elucidate some of signaling events and to assess the behavior and function of mitochondria as well as the induction of respiratory gene accompanied with disease resistance in response to SA. Based on the results obtained from cell imaging and biochemical approaches, this work may contribute to the understanding of the mitochondria-dependent mechanism of the SA-induced biological responses in plants, and provide a new insight into the cellular signaling cascade in SA-mediated defense response.

## Materials and Methods

### Plant Materials and Growth Conditions

The wild-type (WT) (ecotype Columbia), anti-sense line (*aox1a*; N6707) and over-expressing (AOX1a-OE; N6592) seedlings of Arabidopsis AOX1a, *eds4-1* (N3799), and *npr1-1* (N3726) were purchased from NASC. Semi-quantitative RT-PCR was used to determine the effect of T-DNA insertion and over-expression on the transcript levels of *aox1a* and AOX1a-OE lines (S1 Fig.). The transgenic line 43C5 (mito-GFP wild-type; [51]) was obtained from Dr. David C. Logan (St Andrews, UK). The transgenic *NahG*, and *cpr5* mutant lines were kindly provided by Dr. X. Dong (Duke University, Durham, NC). All the Seeds of *Arabidopsis* were sterilized and sown on solid Murashige and Skoog medium containing 0.8% (w/v) agar and 1% (w/v) sucrose (pH 5.8), and incubated for 3 d at 4°C for synchronized germination, and then Petri dishes were placed in a plant growth chamber (Conviron, model E7/2, Canada) with a 16 h light photoperiod (100 μmol photons m^-2^ s^-1^) and 75/80% (light/dark) relative humidity at 23/21°C (light/dark) for 2.5 weeks.

2’, 7’-dichlorodihydrofluorescein diacetate (H_2_DCFDA), MitoTracker Red CMXRos, MitoSOX Red, and Rh123 were obtained from Molecular Probes (Eugene, OR, USA). SA, cyclosporin A (CsA), catalase (CAT, bovine liver), ascorbic acid (AsA), rotenone (5 μM), TTFA (4,4,4-trifluoro-1-(2-thienyl)-1,3-butanedione; 10 μM), antimycin A (5 μM) and potassium cyanide (1 mM) were purchased from Sigma-Aldrich China (Shanghai, China).

### Isolation of Arabidopsis Mitochondria and Assays of Activity of Respiratory Chain Complex III

Mitochondria were isolated and purified by differential centrifugation as described previously, with some modifications [26, 52]. All steps were carried out at 4°C. 400 g of leaves were disrupted with a tissue grinder in 10 mL of cold medium (0.5 M Sucrose, 50 mM Tris, 5 mM EDTA, 0.2% (w/v) BSA, 0.3% (v/v) β-Mercaptoethanol, 0.3% (w/v) PVP-40 and 5 mM Cysteine; pH 7.5). The homogenate was filtered through two layers of miracloth (Calbiochem-Behring) using a syringe. The debris was spun down by centrifugation at 105 g for 10 min and the supernatant was spun at 1600 g for 10 min. Supernatants were collected and centrifuged at 15000 g for 20 min and the pellets resuspended in approximately 1.5 mL of cold medium. The resuspended pellets were centrifuged at 10500 g for 15 min with three replicates, and suspended by acetone and recentrifuged at 15000 g for 10min. Finally, the mitochondria fraction was suspended in assay buffer (300 mM sucrose, 2 mM HEPES, 0.1 mM EDTA; pH 7.4).

The activity of cytochrome reductase was assayed as described by Tiwari *et al*. [53]. The enzyme activity assay was carried out according to the instruction manual of the Tissue Mitochondrial Complex III Assay Kit (Genmed Scientifics Inc., Arlinghton, MA). The assay mixture of the complex III contained mitochondrial protein (10 μg), EDTA, potassium phosphate buffer (pH 7.5), EDTA, bovine serum albumin, and substrates (decylubiquinol and oxidized ferricytochrome c). Under the catalysis of cytochrome reductase (complex III), reduced ubiquinone (UQH_2_) or coenzyme Q (CoQH_2_), as an substrate, was converted into ubiquinone (UQ) or Coenzyme Q (CoQ); as well as ferricytochrome c (ferricyt c^3+^) was reduced to Ferrouscytochrome c (ferrocyt c^2+^). After incubation for 5 min at 30°C, the activity was measured by the increase in absorbance at 550 nm using a molar absorbtion coefficient of 21.84 mmol^-1^ L cm^-1^ for the reduction of ferricytochrome c. The reaction system is as follows,

$${\text{CoQH}}_{\text{2}}\text{+}\;\underset{↓\text{Abs 550nm}}{{\text{2 ferricyt c}}^{\text{3+}}}{\text{+ 2 H}}^{\text{+}}\to \;\text{CoQ +}\;\underset{↑\text{Abs 550nm}}{{\text{2 ferrocyt c}}^{\text{2+}}}{\text{+ 4 H}}^{\text{+}}$$

The complex III antimycin A sensitive enzyme activity was expressed as nmol min^-1^ mg^-1^ protein.

### Respiratory Measurements

Oxygen uptake by isolated mitochondria (adjusted to 0.25 mg protein mL^-1^) was measured at 25°C with a Clark-type oxygen electrode (Hansatech Ltd, Hardwick industrial Estate, King’s Lynn, Norfold, UK) in 1 mL reaction medium (0.3 M sucrose, 5 mM MOPS (pH 7.2), 5 mM KH_2_PO_4_, 2.5 mM MgCL_2_, and 0.1% (w/v) bovine serum albumin). Mitochondrial oxygen consumption was measured with 5 mM malate plus 10 mM glutamate (complex I substrate), or 10 mM succinate (complex II substrate). To establish the discrimination for the alternative and cytochrome oxidases, mitochondria were pre-treated with 1 mM KCN, an inhibitor of complex IV or 20 mM SHAM, an inhibitor of alternative oxidases prior to measurement [54]. The total respitation, CYT-pathway capacity and AOX-pathway capacity were expressed as nmol O_2_ mg^-1^ protein min^-1^.

### Isolation of Arabidopsis Protoplasts and SA Application

The isolation of protoplasts from fourteen-day-old *Arabidopsis* seedlings was performed as described by He *et al*. [55] and Gao *et al*. [56]. Small leaf strips (0.5–1 mm) in the enzyme solution including cellulase R10 and macerozyme R10 (Yakult Honsha, Tokyo, Japan) were vacuum-infiltrated for about 30 min and then incubated in dark for 3 h. After filtration through 75 μm nylon mesh sieves, the crude protoplasts were collected by centrifugation at 100*g* for 3 min. The purified protoplasts were suspended in W5 solution (154 mM NaCl, 125 mM CaCl_2_, 5 mM KCl, 5 mM Glucose, and 1.5 mM MES-KOH, pH 5.6) and the concentration adjusted to between 10^5^ and 10^6^ protoplasts mL^–1^.

After isolation, SA dissolved in water at the indicated concentration was added to 100 μL of protoplast suspension in 96-well plates and incubated for the required periods of time at room temperature.

### Laser Confocal Scanning Microscopy (LCSM)

All microscopic observations were performed using a Zeiss LSM 510 laser confocal scanning microscope (LSM 510/ConfoCor 2, Carl-Zeiss, Jena, Germany). H_2_DCFDA, Rh123, and GFP signals were visualized with excitation at 488 nm and emission at 500–550 nm, and the chloroplast autofluorescence (excitation at 488 nm) was visualized at 650 nm with a long pass filter. MitoTracker Red CMXRos signals were visualized in another detection channel with a 543 nm excitation light and a 565–615 nm band pass filter. All images were taken and analyzed with Zeiss Rel3.2 image processing software (Zeiss, Germany).

### Detection of Mitochondrial ROS Production and Histochemical Staining

An Amplex Red Hydrogen Peroxide Assay Kit was used to measure the concentration of H_2_O_2_ in isolated mitochondria according to the manufacturer’s recommendations. Cellular fractions were added to a 96-well plate with one of the following respiration substrates: 5 mM malate plus 10 mM glutamate (complex I respiratory substrate), or 10 mM succinate (complex II respiratory substrate). The reaction was started with the addition of SA with different concentrations.

After SA treatment, H_2_O_2_ production was determined by detecting the fluorescence of DCF, the product of oxidation of H_2_DCF, as described by Gao *et al*. [56]. The *Arabidopsis* protoplasts were incubated with H_2_DCFDA at a final concentration of 5 μM for 10 min in the dark as described by Yao & Greenberg [57].

The histochemical staining of H_2_O_2_ was performed as described by Orozco-Cardenas & Ryan [58]. Wild-type, *NahG*, *npr1*, *eds4* and *cpr5* leaves were vacuum infiltrated with 1 mg mL^-1^ DAB solution under 25°C.

The production of mitochondrial superoxide was measured using MitoSOX Red staining. MitoSOX Red, a cell-permeable fluorogenic dye, which targets mitochondria selectively, being oxidized by local superoxide, as described by Piacenza *et al*. [59]. The mitochondria (0.5 mg mL^-1^) was isolated from different mutant with altered SA biology (*NahG*, *npr1*, *eds4* and *cpr5* leaves), and incubated with 5 μM MitoSOX Red for 30 min, and then the fluorescence intensity was measured with an LS55 Luminescence Spectrophotometer (PerkinElmer, LS55, UK) at room temperature. The values at 580 nm were used to determine the fluorescence intensity of MitoSOX Red.

### Analysis of Mitochondrial Transmembrane Potential (MTP)

The variation of MTP was measured according to the method described by Gao *et al*. [56]. After SA treatment, the protoplasts were incubated with Rh123 at a final concentration of 2 μg mL^-1^ or were double-stained with Rh123 and MitoTracker Red CMXRos (100 nM) for 30 min at room temperature in darkness. Cells were then harvested, washed, and resuspended homogeneously in W5 solution. The intensity of Rh123 fluorescence was measured by the LS55 spectrometer (excitation 485 nm, emission 505–625 nm). The fluorescence intensity at 526nm was used to determine the relative Rh123 retained in the mitochondria of the protoplasts. The uptake of Rh123 and MitoTracker into cells and into mitochondria was observed under the Zeiss LSM 510.

### Pathogen Maintenance and Pathogen Challenge

*Pst* DC3000 was cultivated on the King’s B liquid medium supplemented with 75 μg mL^-1^ rifampicin at 28°C overnight with shaking until midlog growth phase (optical density at 600 nm, OD_600_ = 0.15) was obtained. To inoculate Arabidopsis with *Pst* DC3000, bacterial cells were retrieved from medium by centrifugation at 3,000*g* for 10 min and resuspended in 10 mM MgCl_2_, and the concentration was adjusted to OD_600_ = 0.01 in 10 mM MgCl_2_. At least 25 plants of Arabidopsis ecotype Col-0 or mutants were inoculated per treatment. Leaves were inoculated with the bacterial suspension of OD_600_ = 0.01 using 1-mL syringes. The inoculated plants were kept in a dew chamber for 16 h at 25°C and 100% relative humidity and then transferred to a growth chamber with a 16-h light/8-h dark regime at 25°C and 80% relative humidity [60]. Bacterial growth was assessed by determining the CFU of 1 g fresh weight of Arabidopsis leaves from five plants through plating appropriate dilutions on King’s B medium containing 75μg mL^-1^ rifampicin [61, 62].

### Total RNA Extraction and Real-time Quantitative RT-PCR

Total RNA was isolated from 2.5-week-old plant seedlings frozen in liquid nitrogen with TRIZOL reagent (Invitrogen) according to the manufacturer’s protocol. Concentration of RNA was determined by measuring OD at 260 nm. First-strand cDNA was synthesized with the SuperScript II First-Strand Synthesis System for RT-PCR (Invitrogen). For real-time quantitative PCR (qRT-PCR) analysis, it was performed using the LightCycler Quick System 350S (Roche Diagnostics K.K.) with SYBR Premix Ex Taq (Takara Bio, Inc.). Each PCR reaction contained 1 × SYBR Premix Ex Taq, 0.2 μM of each primer, and 2 μL of a 1:10 dilution of the cDNA in a final volume of 20 μL. The PCR programme was used: initial denaturation, 95°C, 30 s; PCR, 40 cycles of 95°C, 5 s, 60°C, 20 s with a temperature transition rate of 20°C s^-1^. In melting curve analysis, PCR reactions were denatured at 95°C, annealed at 65°C, then a monitored release of intercalator from PCR products or primer dimmers by an increase to 95°C with a temperature transition rate of 0.1°C s^-1^. Standard curves were created using PCR products by 10-fold serial dilutions. The *AOX1a* gene expression profile was normalized using *actin* mRNA as an internal control. The primers for real-time quantitative PCR are listed in S1 Table.

## Results

### SA Induces ROS Production Mainly in Mitochondria

**Fig 1:**
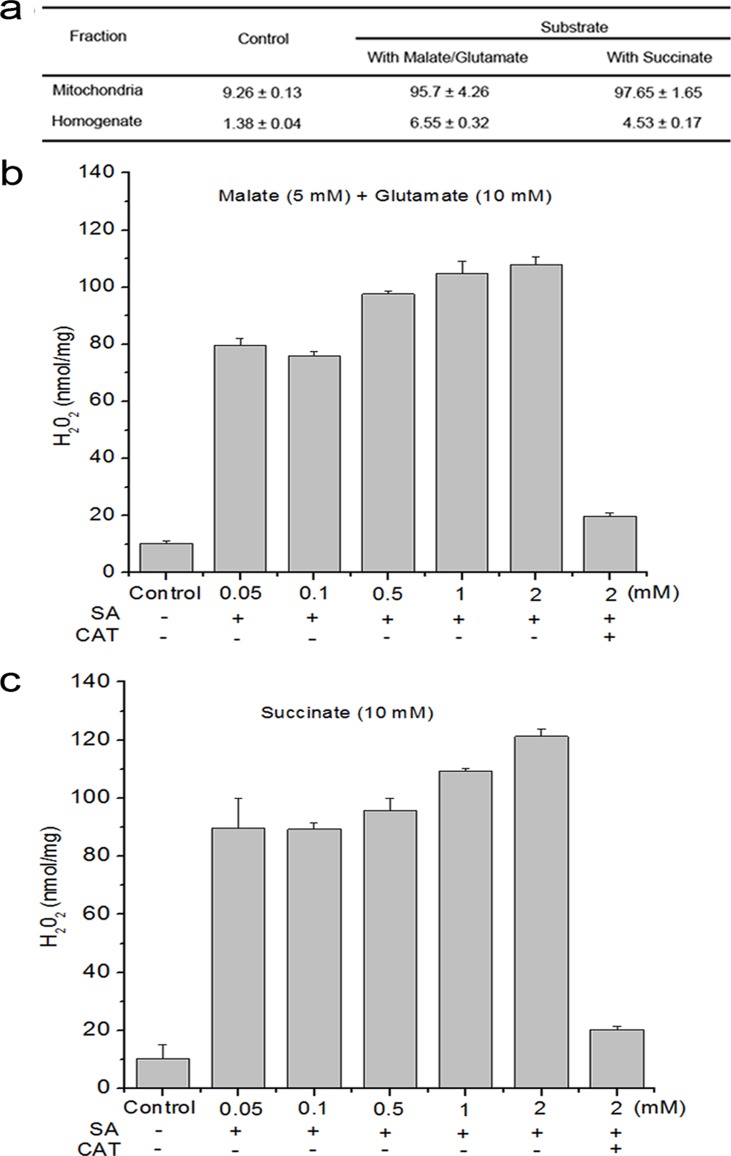
SA induced H_2_O_2_ production in cellular fraction. (**a**) H_2_O_2_ production from isolated mitochondria in the presence of respiration substrates malate plus glutamate (complex I substrate) or succinate (complex II substrate) was measured in the presence of 500 μM SA. (**b**) Dose dependence for SA induced H_2_O_2_ production and accumulation of H_2_O_2_ was reduced by catalase (1000 units mL^-1^) in the presence of respiration substrates malate (5 mM) plus glutamate (10 mM). (**c**) Dose dependence for SA induced H_2_O_2_ production and accumulation of H_2_O_2_ was reduced by catalase in the presence of respiration substrate succinate (10 mM). Data are means ± SD of three different experiments, with similar results.

In order to verify mitochondria are involved in SA-induced ROS production, the *Arabidopsis* leaf homogenate and mitochondria fractions were analyzed for H_2_O_2_ production after the addition of SA. Following the addition of SA in the presence of complex I substrate (malate + glutamate), mitochondria showed an immediate and robust generation of H_2_O_2_, whereas the production of H_2_O_2_ were much lower in homogenate. Similar to the observations above, at the level of complex II substrate (succinate), H_2_O_2_ levels induced by SA in mitochondria were about 24 times as much as the homogenate (Fig. 1a).

To further confirm the involvement of mitochondria in SA-induced H_2_O_2_ production, changes in H_2_O_2_ levels were quantitatively examined by SA ranging from 0 to 1 mM in isolated mitochondria in the presence of complex I or II substrates. There was an increase in H_2_O_2_ production as low as 50 μM SA, and remained increased within 1 mM SA. Conversely, catalase almost completely attenuated this process (Fig. 1b,c).

**Fig 2:**
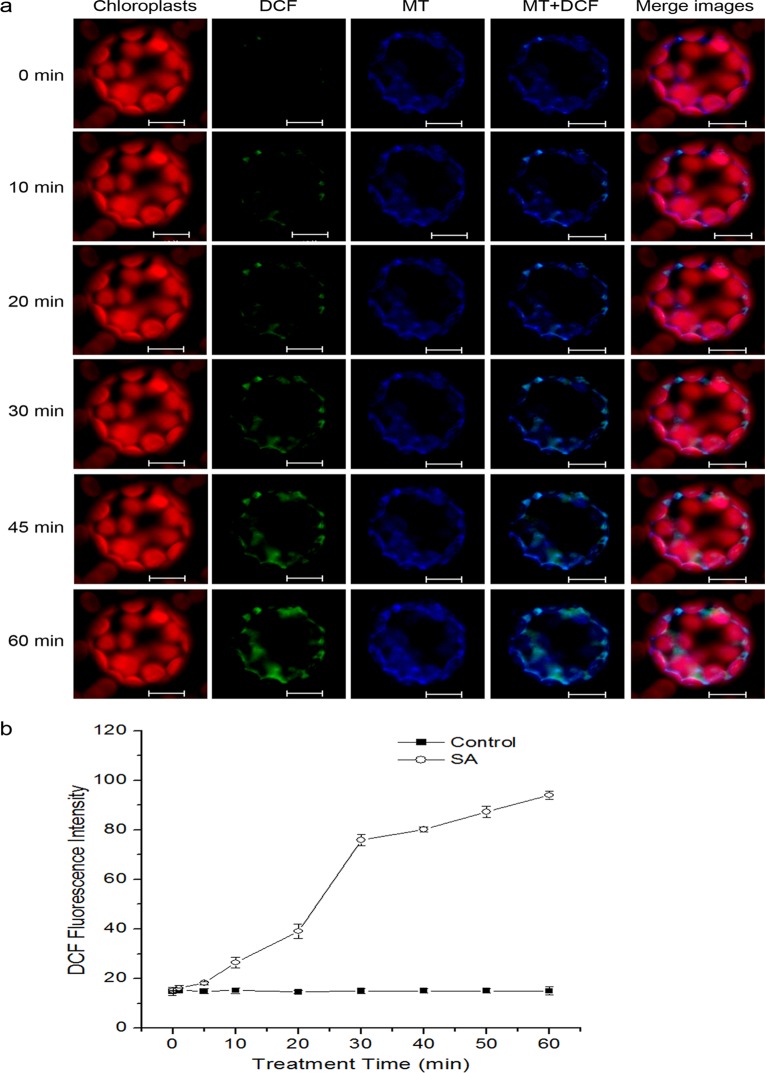
Generation of mtROS under SA treatment in *Arabisopsis* protoplasts. (**a**) The fluorescence imaging of DCF and MitoTracker Red CMXRos in Arabidopsis protoplasts. Protoplasts were treated with or without SA for the indicated time, double-stained with H_2_DCFDA and MitoTracker Red CMXRos (MT), and detected using a LCSM. Scale bars, 10 μm. (**b**) Kinetics graphs of DCF signal intensity of SA-treated isolated mitochondria. Error bars are ±SD values for three replicates.

Next, the protoplasts were used to establish the increased ROS after SA treatment originated from mitochondria. Protoplasts were double-stained with the mitochondria-specific marker MitoTracker Red CMXRos and the ROS probe H_2_DCFDA, which is a cell-permeant dye that contained a mildly thiol-reactive chloromethyl moiety for labeling mitochondria, and a non-fluorescent compound that readily enter cells and form H_2_DCF in the presence of endogenous esterases and H_2_O_2_, respectively [56, 63]. The results showed that protoplasts revealed obvious fluorescent overlap that colocallized in the cytoplasmic areas in which mitochondria were present (Fig. 2a; S2 Fig.). To clarify the results quantitatively, the kinetics of H_2_O_2_ generation was investigated in the isolated mitochondria. At about 10 min after SA treatment, DCF fluorescence intensity began to increase and was significantly boosted at 30 min. Henceforth, the level of H_2_O_2_ kept a slight increase until 60 min (Fig. 2b).

### Endogenous SA Triggers Changed Redox Homeostasis

**Fig 3:**
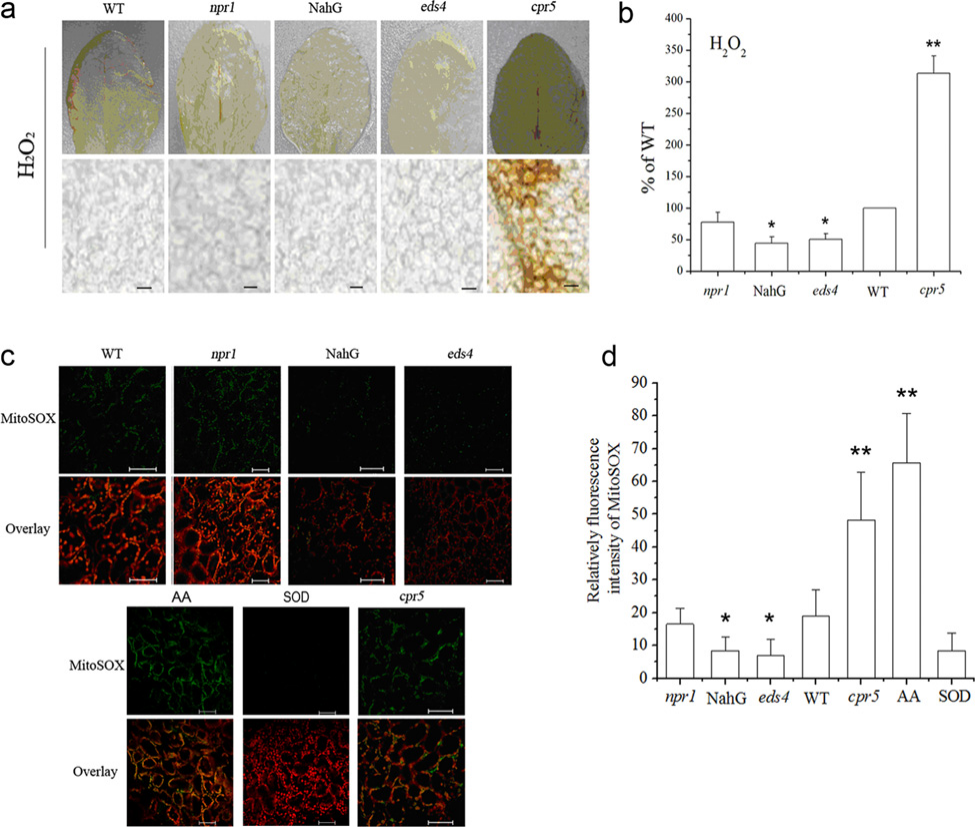
Endogenous SA triggers changed redox homeostasis. (**a**) Foliar levels of H_2_O_2_ in mutants with constitutively high levels of SA and in SA-deficient lines. Scale bars, 300 μm. (**b**) A correlation between SA levels and H_2_O_2_ content in plants. (**c**) The fluorescence imaging of MitoSOX red in wild-type plants and mutants defective in the SA signaling pathway. The fluorescence imaging of MitoSOX red following treatment with the complex III inhibitor antimycin A (AA) and superoxide dismutase (SOD) was shown as positive and negative control, respectively. Chloroplast autofluorescence is false colored red, and MitoSOX Red fluorescence is false colored green. Scale bars, 50 μm. (**d**) Statistical evaluation of fluorescence intensity of Mito SOX in plants. In (**b**) and (**d**), Statistical analysis was performed with Student’s t-test: *, P < 0.05 vs WT; **, P < 0.01 vs WT. Data are means ± SD of three different experiments, with similar results.

The preceding results show that exogenous SA induced mtROS generation (Fig. 2), but an *in vivo* role for SA has not been established. As shown in Fig. 3, the *In situ* detection of leaf H_2_O_2_ was monitored using 3,3’-diaminobenzidine (DAB) staining procedure. The transgenic *NahG* plants expressing a bacterial salicylate hydroxylase, to deplete endogenous SA, and the enhanced disease susceptibility mutant (*eds4*) with reduced accumulation of SA showed much weaker staining and accumulated only 50% of H_2_O_2_ compared with the wild-type. In addition, in *npr1* lines with blocking SA signaling, the accumulation of H_2_O_2_ was similar to that of wild-type, without increased accumulation of H_2_O_2_. However, mutant with constitutive accumulation of SA (*cpr5*) exhibited enhanced accumulation of H_2_O_2_ (Fig. 3a,b).

The cell-permeable fluorogenic probe MitoSOX Red targets mitochondria selectively, being oxidized by local superoxide [59]. Similar to the above results, endogenous contents of SA were correlated to the MitoSOX Red fluorescence (Fig. 3c,d).

### The Effect of SA on Mitochondrial Respiration in *Arabidopsis*

**Fig 4:**
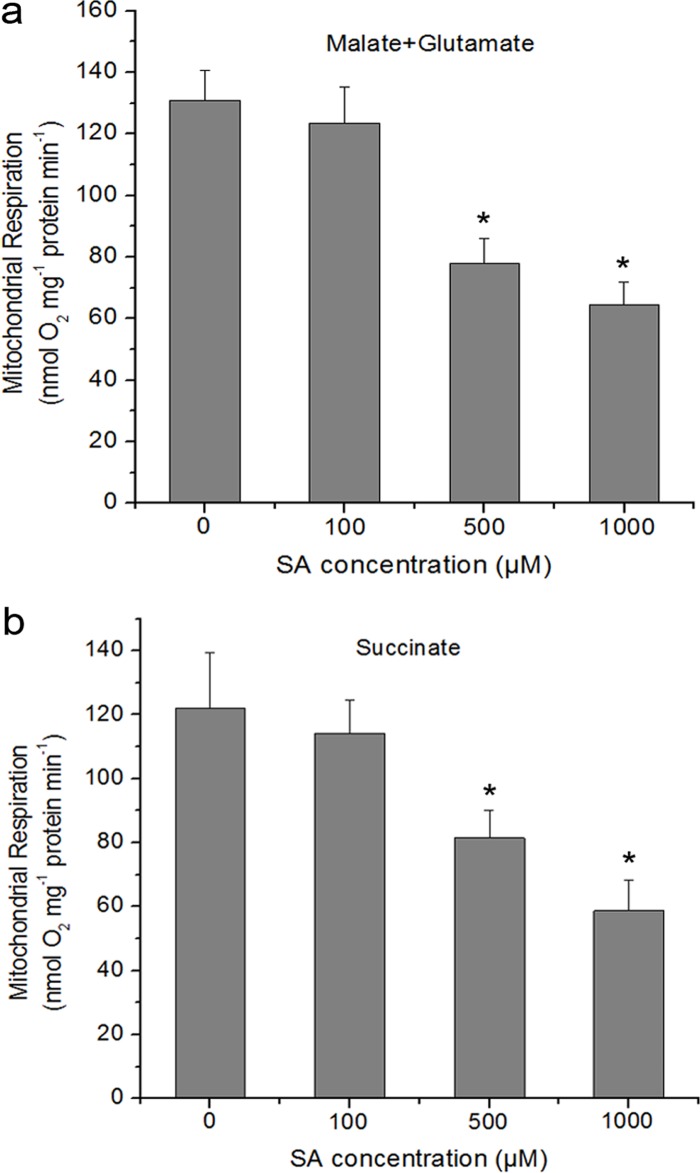
Respiratory parameters of the isolated mitochondria in response to different concentrations of SA. Respiration rates (nmol O_2_ mg^−1^ protein min^−1^) were measured using 5 mM malate plus 10 mM glutamate (**a**) or 10 mM succinate (**b**) as respiratory substrates. SA was added to the reaction vessel to the final concentration indicated for each histogram. Asterisks indicate significant differences to 0 μM SA (Student’s t-test, P <0.05). Each value is the mean ± SD of four replicates.

To determine whether the production of mtROS is associated with respiratory functions in isolated mitochondria or not, the respiratory characteristics of isolated mitochondria were analyzed treated with or without SA by measuring oxygen consumption [64]. The total respiration of isolated mitochondria was measured with complex I or II substrates. As shown in Fig. 4a, using malate/glutamate as the electron donor, treatment of the isolated mitochondria with SA decreased respiration rate with a dose dependent. The respiration rate decreased by 41% and 50.4%, respectively, as compared to control after 0.5 and 1 mM SA treatment. The similar results of respiratory property were observed with succinate provided as substrate (Fig. 4b).

**Fig 5:**
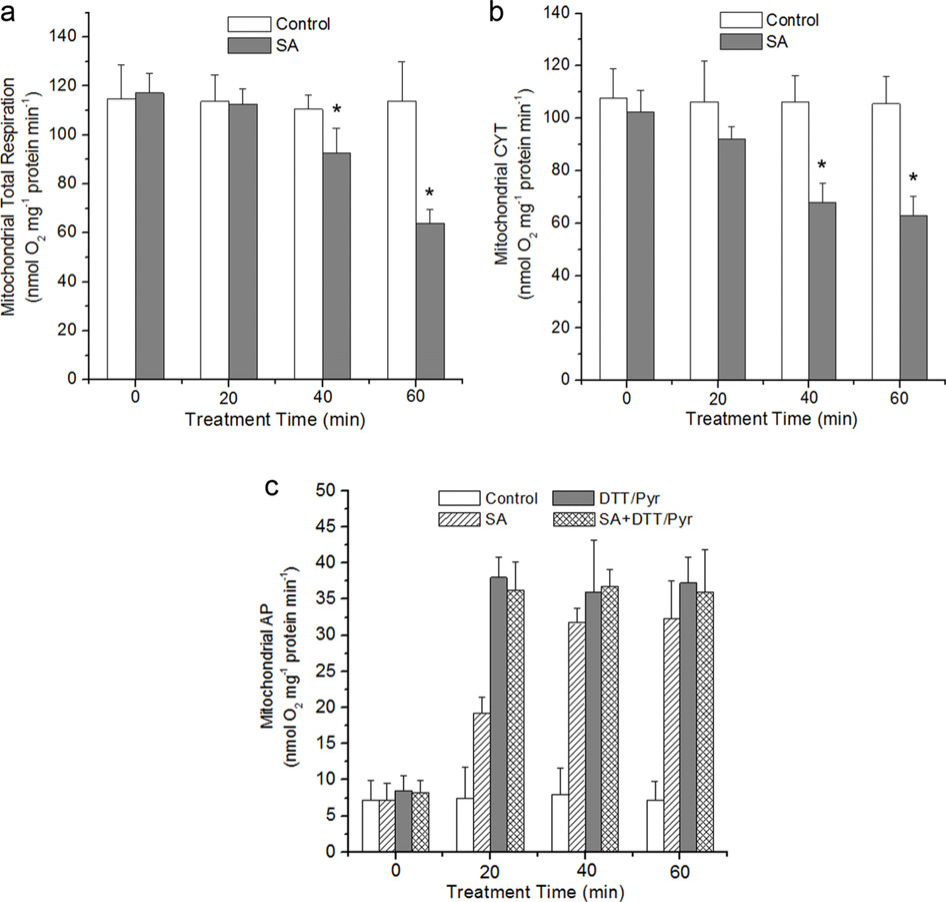
Time course of respiration of isolated mitochondria under 500 μM SA. The changes of mitochondrial respiration, CYT-pathway and alternative respiration pathway (AP) capacities in wild-type under SA treatment were shown in (**a**), (**b**) and (**c**), respectively. To establish the discrimination for cytochrome and alternative oxidases, mitochondria were pre-treated with 1 mM KCN, an inhibitor of complex IV or 20 mM SHAM, an inhibitor of alternative oxidases prior to measurement. In (**c**), the DTT (10 mM) and pyruvate (Pyr, 1 mM) were supplemented to ensure the biochemical activation of AOX protein. Asterisks indicate significant differences to control (Student’s t-test, P <0.05). Data are means ± SD of four different experiments, with similar results.

Plant mitochondria are unique in that they possess a bifurcated electron transport chain, including cyanide-insensitive alternative respiratory pathway (AP) and salicylhyroxamic acid-insensitive cytochrome pathway (CYT) [65]. To examine the above respiration changes more closely, a time-course experiment was performed under 0.5 mM SA treatment. Under SA treatment, the mitochondrial total respiration exhibited a remarkable decrease. At 40 and 60 min, the respiration rate decreased by 16% and 44%, respectively (Fig. 5a). The capacity of CYT pathway decreased whereas that of AP showed an obvious increase as compared to control (Fig. 5b,c). The CYT was reduced by 39% and the AP respiration was increased by 345% up to 60 min.

### SA Inhibits the Activity of Mitochondrial Respiratory Complex III

**Fig 6:**
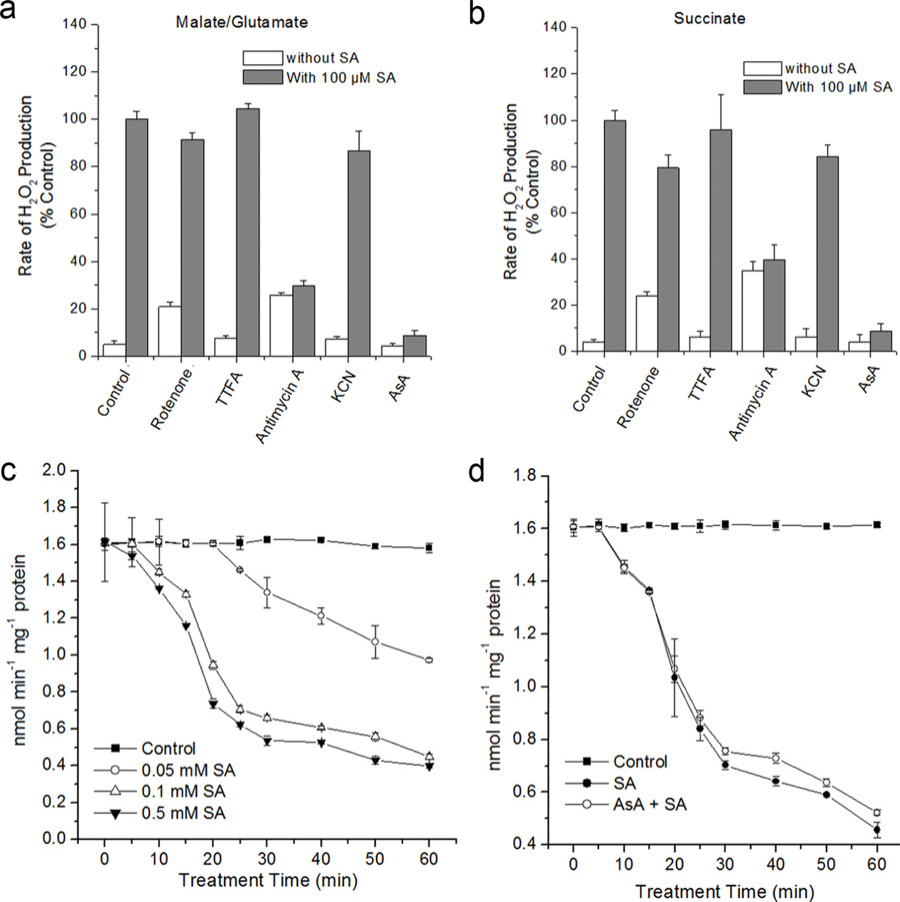
Effects of SA on the mitochondrial ROS and changes of respiratory complex activity. (**a**) and (**b**), Mitochondria were isolated and stimulated in the presence of complex I (malate + glutamate) or II (succinate) substrates, and H_2_O_2_ production was measured in the absence or presence of SA. H_2_O_2_ production in mitochondria stimulated with malate/glutamate or succinate as respiration substrates in the presence of SA was considered 100%. Asterisks indicate significant differences to control under SA treatment (Student’s t-test, P <0.05). (**c**) and (**d**), The activity of complex III in SA-treated isolated mitochondria with or without pretreatment of 1 mM AsA. Error bars are ± SD values for three replicates.

SA treatment induced mtROS generation and reduced the respiratory rate with the complex I and complex II substrates (Figs. 2 and 4), possibly mitochondrial components which are important for electron transport were implicated in this process. To test this hypothesis, SA-induced H_2_O_2_ production in isolated mitochondria was determined in the presence of inhibitors of the electron transport chain. ROS generation is assayed in the presence of complex I (malate + glutamate) or II (succinate) substrates, which acts to feed electrons into the ETC at the level of complex I via NADH or directly to complex II, respectively. As shown in Fig. 6 a and b, mitochondria stimulated by malate/glutamate or succinate produced a significant increase in H_2_O_2_ generation under SA, whereas exogenous AsA almost completely attenuated this process. Rotenone and antimycin A (a specific inhibitor of complex I and III, respectively) produced a significant rate of H_2_O_2_ generation at the absent of SA. However, SA co-administration with antimycin A showed rates similar to those observed with antimycin A alone. Further, the decreases in rates of H_2_O_2_ production were not observed with complex I, II and IV inhibitors under SA treatment. Rotenone also decreased H_2_O_2_ generation via succinate, but to a lesser extent than antimycin A (Fig. 6b). These data indicate that complex III is centrally involved in H_2_O_2_ generation by SA.

To further confirm the role of complex III in modulating ROS generation, the activity of complex III was determined under SA treatment. Fig. 6c showed that complex III activity exhibited a time- and concentration-dependent decrease. As early as 10 min after SA treatment, the activity of complex III obviously began to decrease, which is prior to the generation of mtROS (Fig. 2). Moreover, pre-treatment of AsA failed to inhibit the decline in the activity of complex III (Fig. 6d), indicating that complex III might be as a direct target for SA treatment.

### Relationship between Mitochondrial Morphology and SA-Induced mtROS in *Arabidopsis*

**Fig 7:**
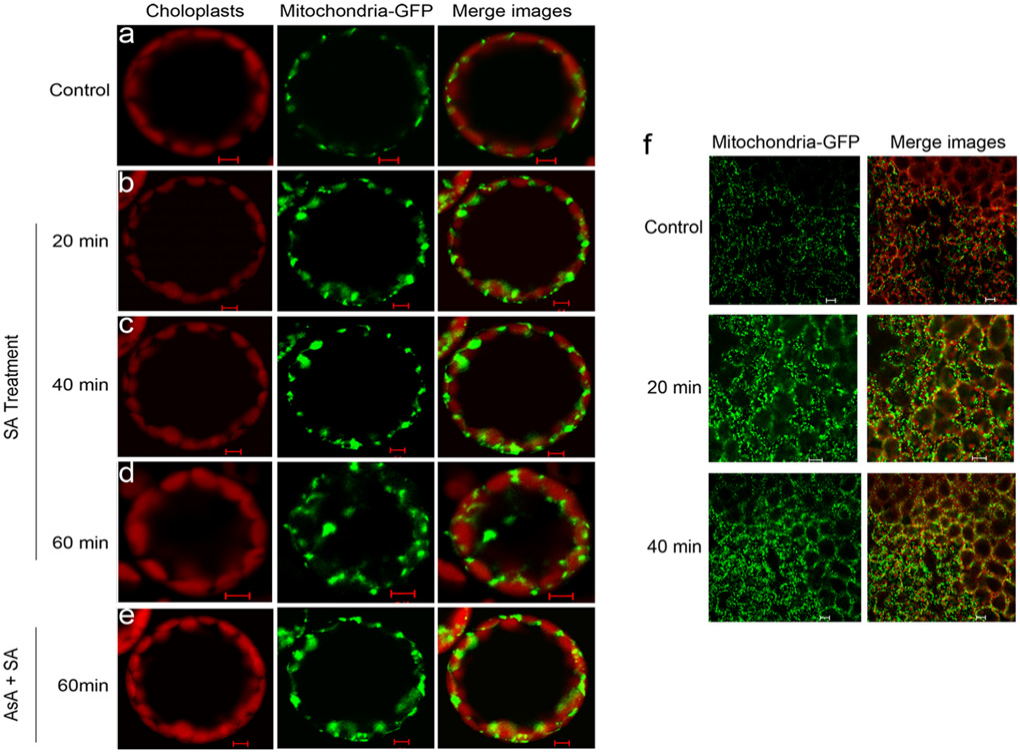
Abnormal Mitochondrial morphology after treatment with SA. Confocal images of mito-GFP mesophyll protoplasts were taken in the absence of 500 μM SA for 1 h (**a**) or in the presence of 500 μM SA for 20 min (**b**), 40 min (**c**) or 60 min (**d**). Confocal images of mito-GFP mesophyll protoplasts which were pre-incubated with AsA at 1 mM final concentration for 30 min and treated with SA for 1 h were also taken (**e**). Scale bars, 10 μm. (**f**) Micrographs of mitochondrial morphology in *Arabidopsis* leaf tissue during SA treatment. Leaves of at least five plants were examined, and the experiment was performed three times. Chloroplast autofluorescence is false colored red, and GFP fluorescence is false colored green. Scale bars, 20 μm.

To establish if alterations in mitochondrial morphology were mtROS-specific, Arabidopsis mesophyll protoplasts expressing mito-GFP (43C5) were incubated with SA. In control protoplasts and leaves, mitochondria, retained as filamentous structures, were restricted to a narrow band of cytoplasm surrounding the plasma membrane over 60 min (Fig. 7a). After 20–40 min of SA treatment, mitochondria began to show an aggregated distribution, with tens of mitochondria arranged into clusters and the plan areas of individual mitochondria increased or became swollen (Fig. 7b,c). By 60 min, mitochondria showed a more irregular clumped or clustered morphology within the cytoplasm (Fig. 7d). The addition of ROS scavenger ascorbic acid (AsA) before SA treatment led to markedly reduced changes in mitochondrial morphology, indicating that the aggregation of mitochondria is attributable to ROS accumulation (Fig. 7e). Like mitochondria in SA-treated protoplasts, mitochondria from SA-treated leaves showed an increase in plan area at the indicated times (Fig. 7f).

### Mitochondria Undergo Member Potential Changes in Response to SA

**Fig 8:**
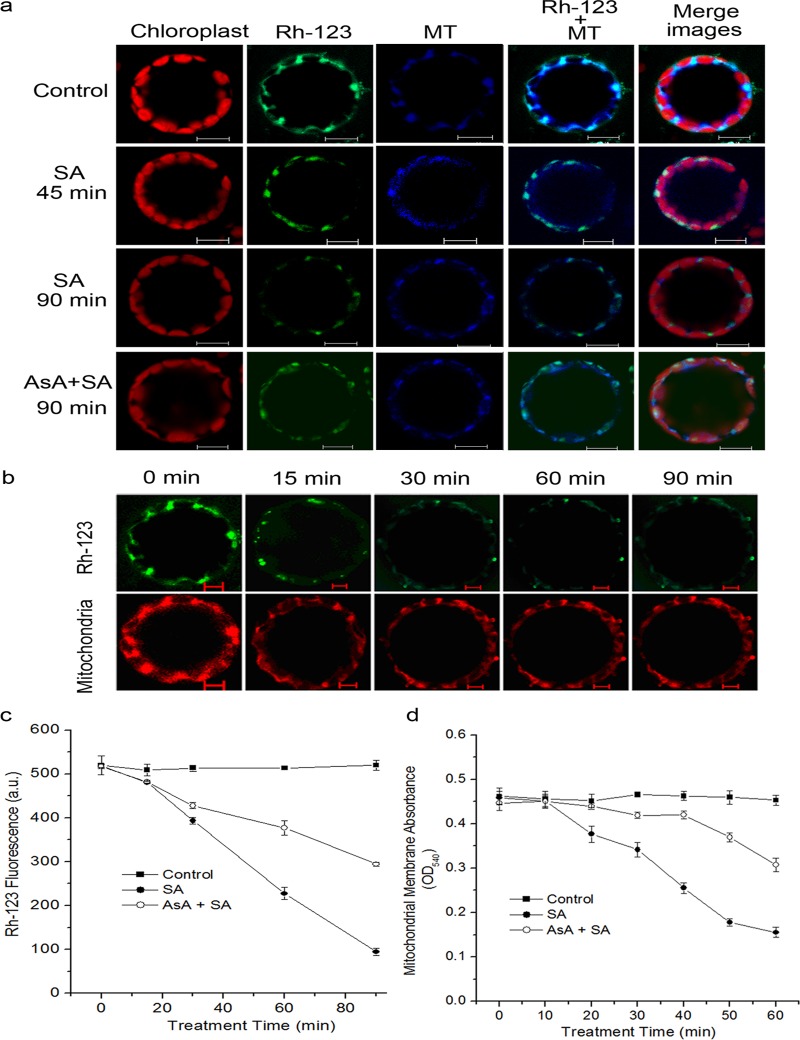
Mitochondrial depolarization induced by SA treatment. (**a**) Protoplasts were pre-incubated with or without AsA at 1 mM final concentration for 30 min, and were then untreated for 1.5 h or treated with 500 μM SA for 1.5 h. Samples were double stained with Rh-123 and MitoTracker Red CMXRos and observed with a LCSM. (**b**) Time course of the fluorescence intensity of Rh-123 or MitoTracker within 1.5 h after treatment with SA. Scale bars, 10 μm. (**c**) Analysis of mitochondrial MTP in SA-treated protoplasts using fluorescence probe Rh-123. (**d**) Mitochondrial swelling indicated by the changes in absorbance at 540 nm (OD_540_). In (**c**) and (**d**), each value is the mean ± SD of three replicates.

As described above, a rapid and consistent change in mitochondrial morphology was observed in response to SA. We further examined the changes of mitochondrial transmemberane potential (MTP) to explore whether the observed changes in mitochondrial morphology were related to MTP disruption in this type of ROS generation event. The variations of MTP were determined by rhodamine 123 (Rh123), which is a specific fluorescent probe for monitoring active mitochondria, and the uptake into the mitochondrial matrix depends on the MTP [66]. As shown in Fig. 8, the mitochondrial specific marker MitoTracker Red CMXRos was used to confirm Rh123 was mostly localized to mitochondria. When the control protoplasts were kept in dim light for 1.5 h, they were stained extensively with Rh123, and the fluorescence of which co-localized with MitoTracker, determining the specificity of Rh123 for mitochondria (Fig. 8a; S3 Fig.). After addition of SA to the protoplasts, they showed a time-dependent decrease in MTP compared with the control (Fig. 8b). In the first 15 min, dye accumulation began to decrease and continued to decrease at 45–90 min (Fig. 8c). At 45 min, only the clusters of mitochondria could be stained. Futher, at 90 min, a very low intensity was observed (Fig. 8a,b). However, pre-incubation of AsA to SA-treated protoplasts caused to effectively retard the MTP decrease (Fig. 8a,c).

The state of MTP in the *Arabidopsis* cells treated with SA was assayed by measuring the mitochondrial swelling, which were measured as a rapid absorbance loss at 540 nm (OD_540_, [67, 68]). The isolated mitochondrial with SA treatment caused a progressive decrease in the absorbance at 540 nm. At 60 min after SA treatment, the absorbance at 540 nm decreased to 35% of the control sample, and pre-treatment with AsA effectively delayed and inhibited the absorbance decrease (Fig. 8d).

### Alternative Oxidase (AOX) Gene Expression Is Up-Regulated in Response to SA, and the AOX Induction Is Dependent on NPR1

**Fig 9:**
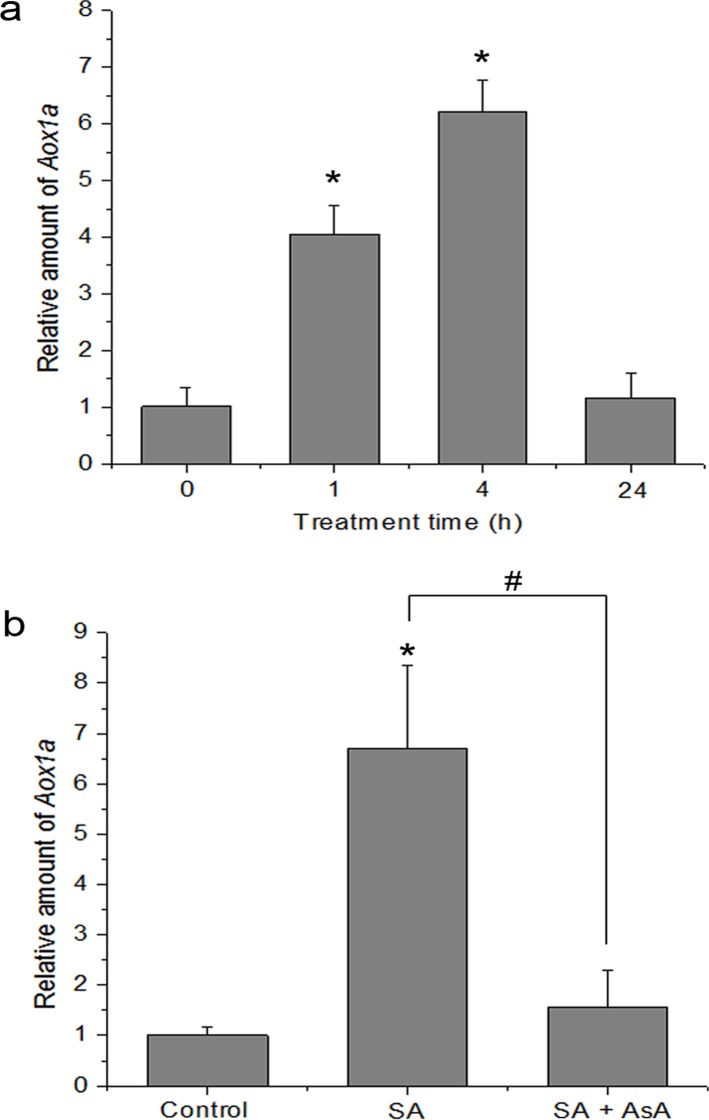
Effect of SA on the expression of *Aox1a* transcript. (**a**) Real-time quantitative RT-PCR of *Aox1a* from control (0 h) and seedlings treated with 500 μM SA for 1, 4 and 24 h. Statistical analysis was performed with Student’s t-test: *, P < 0.05 vs 0 h. (**b**) Effect of antioxidant AsA on SA-induced AOX1a gene expression. The seedlings were treated with 500 μM SA for 4 h. Statistical analysis was performed with Student’s t-test: *, P < 0.05 vs control. #, P < 0.05 vs SA. Data are means ± SD of three different experiments, with similar results.

Inhibition of mitochondrial respiration by SA has been shown to be accompanied by induction of alternative oxidase (AOX) [43, 48]. Therefore, AOX induction is regarded as a marker for mitochondrial oxidative stress. In the AOX family, AOX1a is an important member and often dramatically induced at the transcript level by a variety of stresses [44], while other gene family members display tissue or developmental specificity in their expression [46]. The induction of the transcript encoding *AOX1a* was the highest level 4 h after SA treatment. The *AOX1a* transcript abundance decreased between 4 and 24 h after SA treatment and had decreased nearly to control levels after 24 h (Fig. 9a). However, accumulation of *AOX1a* mRNA upon SA treatment was arrested by pre-treatment with antioxidant AsA (Fig. 9b).

NPR1, as a regulator protein, is required in development of induced resistance induced by SA or pathogen infection [69, 70]. It is necessary to evaluate whether SA-induced *AOX1a* gene expression is dependent on NPR1. As shown in S4a Fig., the *npr1* mutant exhibited reduced expression of *AOX1a* transcript compared with WT plants under SA treatment. Further, the interaction of H_2_O_2_ and AOX1a in *npr1* mutant was evaluated. As speculated, the level of *AOX1a* was increased in response to H_2_O_2_ in a concentration-dependent manner in wild-type *in vitro* (S4b Fig.). *AOX1a* was activated at concentrations of H_2_O_2_ as low as 100 μM, and significantly enhanced as H_2_O_2_ dose increased. At 5 mM, H_2_O_2_ clearly promoted *AOX1a* expression compared with the control (0 mM), whereas the induction of *AOX1a* mRNA was markedly reduced in the *npr1* mutant. These data indicated that SA induces *AOX1a* transcript expression through an NPR1-dependent signaling pathway.

### AOX Contributes to Defense Response to Pathogen Attack

To confirm whether AOX play a crucial role in resistance induced by SA, the response to *Pst* DC3000 upon pretreatment with SA in wild-type plants was determined. Leaves from wild-type plants were pretreated with water (Control) or SA and subsequently inoculated with *Pst* DC3000. Most leaves without SA treatment (Control) exhibited light yellow at 3 days after challenge inoculation, and finally wilted and died 5 days post-inoculation. In contrast, pretreatment with SA, Arabidopsis plants showed no visible symptoms at 3 days post-inoculation. As expected, SA-treated wild-type leaves also developed significant reduction of bacterial growth compared with the control. However, pretreatment with AOX inhibitor such as salicylhydroxamic acid (SHAM) caused an increase in bacterial growth at 3 d and 5 d after challenge inoculation (S5a,b Fig.). Interestingly, AsA-pretreatment also effectively interdicted these effects (S5a Fig.).

**Fig 10:**
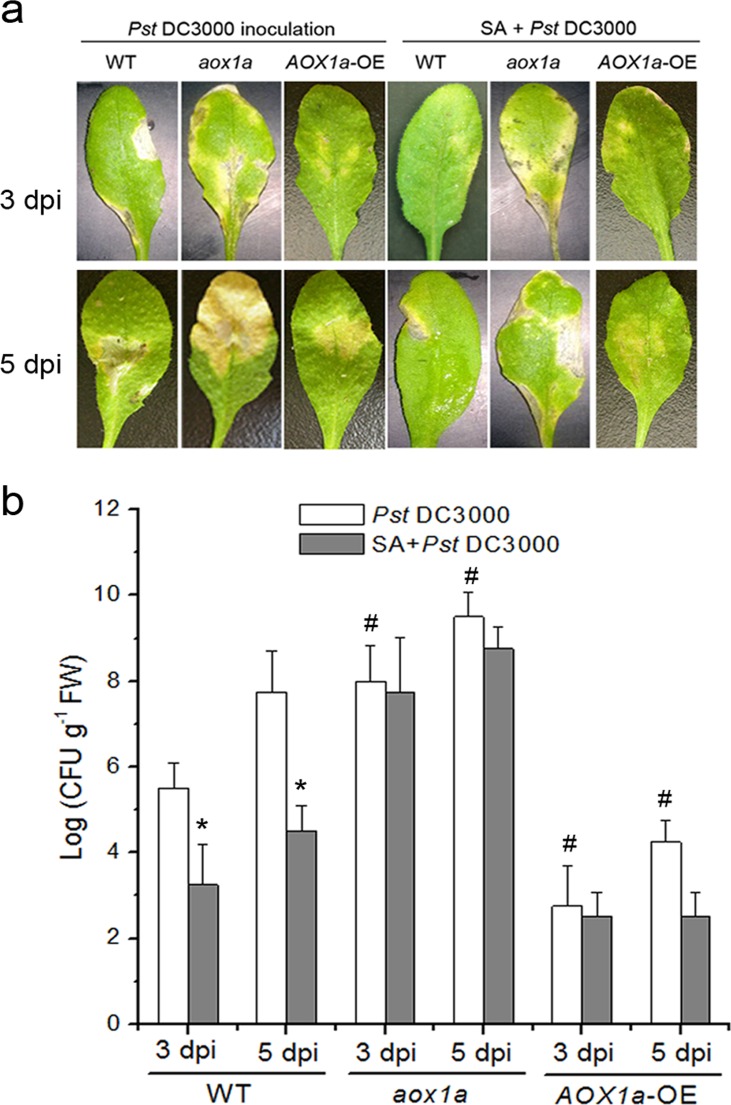
Role of AOX in SA-induced disease development in Arabidopsis. (**a**) The necrotic lesions on representative Arabidopsis leaves at 3 or 5 days after infected by *Pst* DC3000 in wild-type, *aox1a* mutant and *AOX1a-*OE plants. The plants were sprayed with either water or SA, and then inoculated with *Pst* DC3000. The infection was observed 3 and 5 days after inoculation. (**b**) *Pst* DC3000 growth analysis in detached leaves of wild-type, *aox1a* mutant and *AOX1a-*OE plants. FW, fresh weight. Asterisks indicate significant differences to control (student’s t-test: *p < 0.05), and #, P < 0.05 vs *Pst* DC3000 in wild-type. Data are means ± SD of four different experiments, with similar results.

We next determined the disease resistance in wild-type, *aox1a* mutant and *AOX1a*-overexpressing mutant (*AOX1a*-OE) plants. Leaves from wild-type or mutant plants were pretreated with water or SA and subsequently inoculated with *Pst* DC3000. As shown in Fig. 10a,b, *AOX1a*-OE plant leaves displayed more resistance and developed significant reduction of bacterial growth with respect to leaves treated with wild-type. SA-pretreated wild-type leaves developed significant reduction of bacterial growth, whereas the *aox1a* mutant was significantly more susceptible to *Pst* DC3000 and also failed to develop resistance by SA.

## Discussion

This study was an attempt to understand the role of mitochondria in response to SA in detail, through the investigation of a cascade of phenomena in SA-exposed mitochondria or protoplasts. Our work clearly showed that mitochondria are a major source of ROS generation induced by SA; the behavior and function of mitochondria as well as the induction of respiratory gene is closely dependent upon mtROS. SA caused a change from gross mitochondrial morphology to function. Such SA-induced mitochondrial disturbance may be a broadly employed strategy, and increased mtROS by SA may contribute to the protection of plants against further pathogen attack.

The mtETC system generates oxygen radicals through electron leaks as substrates are metabolized [24–26]. In this study, in the absence of respiration substrates, SA-induced ROS generation was highest in the mitochondrial fraction, which might be explained by the fact that mitochondria kept on reducing equivalents following isolation, and mitochondria were able to undergo respiration during this time (Fig. 1). However, the addition of respiration substrates at the level of complex I (malate/glutamate) or complex II (succinate), which acts to feed electrons into the ETC at the level of complex I via NADH or directly to complex II, respectively, generated significant H_2_O_2_ in the mitochondria compared with other cell compartments. This dependent of SA-induced ROS generation in the present of respiration substrates suggests the involvement of mtETC. On the basis of protoplasts double-stained with DCF and MitoTracker Red CMXRos, the results showed obvious fluorescent overlap that colocalized in the cytoplasmic areas in which mitochondria were present (Fig. 2). This phenomenon pointed to the question how the production of mtROS occurred under SA treatment. Previous study with SA in tobacco cells has showed respiration inhibitions [35, 43]. SA significantly impacts on mitochondrial function in a dose dependent manner, with inhibition of electron flow and alteration of respiration rate [36]. Here, a clear inhibition of respiration in a dose dependent manner was observed (Fig. 4) and the malate/glutamate- or succinate-dependent and CYT-pathway capacities were also inhibited in isolated mitochondria under SA stress (Fig. 5). This inhibition of respiration may be possibly due to the inhibition of electron flow [71]. In addition, Maxwell *et al*. [72] found that SA caused mitochondrial dysfunction, which mimicked the specific inhibition of mitochondrial electron transport caused by antimycin A. The most conspicuous effect observed under SA treatment is the rapid inhibition of electron flow and respiratory rate [35, 36, 73]. So it is of be interest to identify the target of SA action among the mitochondrial complexes. Our results pinpointed cytochrome c reductase (complex III) possible as the main target for SA in the mitochondrial ETC (Fig. 6). Under normal conditions, the respiratory I or II substrates can feed electrons directly and flow into the ubiquinone cycle and transfer them to complex III [71]. Under SA treatment, the activity of complex III was affected by SA in a time- and concentration-dependent manner, which could not be alleviated by eliminating ROS. Therefore, the mtROS are generated through electron leaks, depending on inhibition of specific sites in the ETC or the reduction state of the ETC components, as substrates are metabolized [27], indicating that SA might act directly on the complex.

As an indicator of mitochondrial activity, mitochondrial morphology is related to physiological function and energy metabolism [33, 74]. In plants, the ROS-dependent disruption of morphology and collapse of MPT has been described [38, 56, 68, 75, 76] and the MPT is characterized by several features, including the loss of Δψ_m_ and the formation of the permeability transition pore (PTP) [77, 78]. Here, our results showed that SA caused a rapid in gross mitochondria morphology on a time-scale. Using the *Arabidopsis* transformants expressing mito-GFP (43C5), mitochondria under treatment with SA for 60 min showed an aberrant phenotype, including an increase in the aggregated distribution and an arrangement into clusters (Fig. 7), which is consistent with the findings of [38]. Accompanied with this alteration, decreased in MPT was observed under SA treatment (Fig. 8). Strikingly, by pre-incubating protoplasts with the antioxidant AsA, the aberrant phenotype and MTP loss could be effectively prevented (Fig. 7d,e; [8]). In addition, an inhibitor of the mitochondrial PTP, cyclosporine A (CsA), also led to effectively retard the MTP decrease induced by SA (S6 Fig.; [77]). Our data demonstrated that SA-induced mtROS are thought to be critical factors promoting mitochondrial morphology transition, including PTP opening.

AOX, An alternative oxidase in plant mitochondria, can accept electrons from ubiquinol, dissipate the redox potential as heat and protect against oxidative damage. Rhoads & Mclntosh [79] and Norman *et al*. [36] found that SA induces AOX because AOX expression appears to increase in response to discruptions in respiratory homeostasis induced by diverse stress [80]. Hence, AOX may represent an excellent “reporter gene” to evaluate mitochondrial function. An effective means to induce expression of AOX is by artificial chemical inhibition of the cytochrome pathway by compound such as AA [65]. This suggests that when the capacity of CYT is altered, it signals coordinate changes in the capacity for AOX respiration. Here, mitochondrial function could modulate this respiratory gene under SA treatment. Accompanying the inhibition of CYT, an up-regulation of the expression of *AOX1a* transcript was observed in response to SA, and this resulted in a large increase in AOX-pathway capacity (Figs. 5 and 9). However, accumulation of *AOX* mRNA was inhibited by the antioxidant AsA, indicating that ROS from mitochondria could be as an important intermediary in gene induction by SA. During HR, mitochondria are proposed to be a death integrator [7, 20, 30]. Accompanying mitochondrial morphology transition, mitochondria can perceive and integrate signals to influence the defense responses for further pathogen attack, such as SAR. In this paper, upon pathogen attack, *AOX1a*-OE plants displayed enhanced disease resistance compared with wild-type, whereas *aox1a* mutant was significantly more susceptible to pathogen and also failed to develop resistance by SA. AsA-pretreatment also effectively interdicted the defense response (Fig. 10; S5 Fig.). In addition, Vellosillo *et al*. [81] reported the role of mitochondria in plant defense response using *nonresponding to oxylipins* (*noxy*) mutants, and found that alteration of mitochondrial shape and distribution caused functional dysfunction, and reduced SA up-regulation of gene expression and enhanced susceptibility. From this point, mitochondria play an active role, and it may represent an important intermediate between the perception of stress and downstream response such as the induction of defense gene expression [20, 21, 81].

Recently, NPR1, as a regulator protein, is required in development of induced resistance induced by SA or pathogen infection [69, 70]. It is necessary to evaluate whether SA-induced *AOX1a* gene expression is dependent on NPR1. As shown in S4a Fig., the *npr1* mutant exhibited reduced expression of *AOX1a* transcript compared with WT plants under SA treatment. Further, we evaluated the interaction of H_2_O_2_ and AOX1a in *npr1* mutant. As speculated, the level of *AOX1a* was increased in response to H_2_O_2_ in a concentration-dependent manner in wild-type *in vitro* (S4b Fig.). *AOX1a* was activated at concentrations of H_2_O_2_ as low as 100 μM, and significantly enhanced as H_2_O_2_ dose increased. At 5 mM, H_2_O_2_ clearly promoted *AOX1a* expression compared with the control (0 mM), whereas the induction of *AOX1a* mRNA was markedly reduced in the *npr1* mutant. These data indicated that SA induces *AOX1a* transcript expression through an NPR1-dependent signaling pathway. However, the concrete interaction between NPR1 and this respiratory gene AOX1a in SA-induced signaling pathway is still needed to be study in future work.

**Fig 11:**
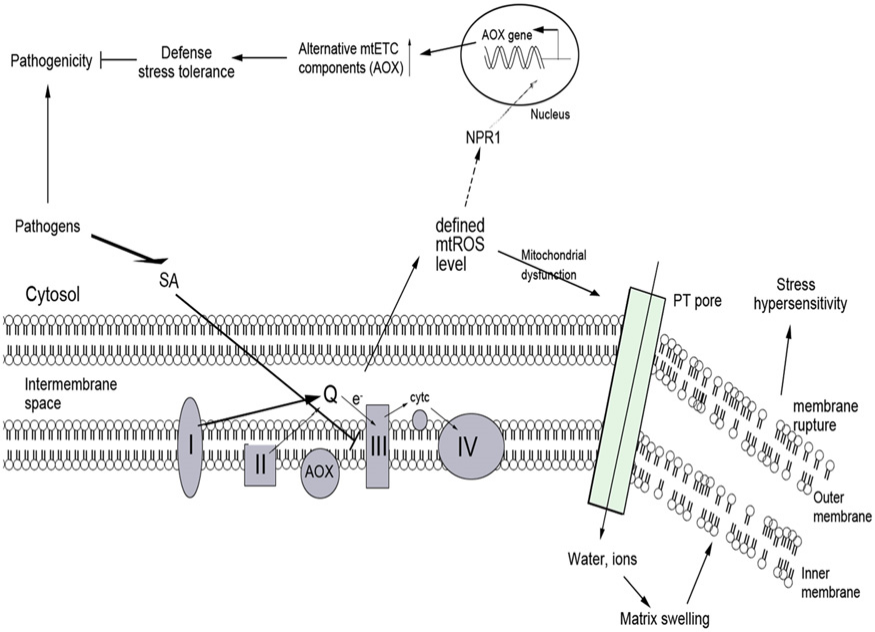
Hypothetical model for mitochondrial dysfunction and AOX defense in SA-induced ROS signal pathway. SA blocks electron flow, and inhibits the function of mtETC components such as CYT pathway, resulting in increased mtROS. On one hand, mtROS causes mitochondrial dysfunction associated with morphology transition and depolarization of membrane potential. On the other, the expression of the unique alternative components of mtETC (AOX) appears to increase in response to discruptions in respiratory homeostasis, and further, AOX contributes to defense response to invading pathogens in whole plants.

In conclusion, our data showed that the mtROS generation, mitochondrial morphology transition, depolarization of mitochondrial membrane potential, the modulation of mitochondrial respiratory gene, and induction of defense response, in that order, played an essential role in SA-mediated signaling pathway. Accordingly, a potential cascade of cellular events in SA-mediated signaling pathway was proposed (Fig. 11). Our study contributed to the understanding of the mitochondria-dependent mechanism of the SA-induced biological response in plants, and might provide a new insight into the cellular signaling cascade in SA-mediated defense response.

## Supporting Information

S1 Fig Characterization of *aox1a* mutant and AOX1a-OE line using semi-quantitative RT-PCR. The total RNAs were extracted from the leaves of 2-week-old Arabidopsis wild-type, *aox1a* mutant and AOX1a-OE seedlings and analyze the transcripts by the semi-quantitative RT-PCR, and the *Actin2* was analyzed as standard.
(TIF)

S2 Fig Change of Pearson’s correlation coefficient (PCC) between DCF and MitoTracker Red CMXRos fluorescence in Arabidopsis protoplasts in Fig. 2a with Image-Pro Plus (IPP) software. Protoplasts were treated with or without SA for the indicated time, double-stained with H_2_DCFDA and MitoTracker Red CMXRos (MT), and detected using a LCSM. The co-localization between DCF and MitoTracker Red CMXRos fluorescence was examined using IPP software. Data are means ± SD of three different experiments, with similar results.
(TIF)

S3 Fig Change of Pearson’s correlation coefficient (PCC) between Rh123 and MitoTracker Red CMXRos fluorescence in Arabidopsis protoplasts in Fig. 8a with Image-Pro Plus (IPP) software. Protoplasts were pre-incubated with or without AsA at 1 mM final concentration for 30 min, and were then untreated for 1.5 h or treated with SA for 1.5 h. Samples were double stained with Rh-123 and MitoTracker Red CMXRos and observed with a LCSM. The co-localization between Rh123 and MitoTracker Red CMXRos fluorescence was examined using IPP software. Data are means ± SD of three different experiments, with similar results.
(TIF)

S4 Fig Effect of SA on the expression of *Aox1a* transcript in *npr1* mutant. (**a**) Real-time quantitative RT-PCR of *Aox1a* in WT and *npr1* mutant plants from control (0 h) and seedlings treated with 500 μM SA for 1, 4 and 24 h. Statistical analysis was performed with Student’s t-test: *, P < 0.05 vs 0 h. (**b**) Effect of H_2_O_2_ on *AOX1a* gene expression in WT and *npr1* mutant. Seedlings were treated with increasing concentrations of H_2_O_2_ (0–5 mM), and the expression of *AOX1a* was analyzed by real-time PCR. Arabidopsis *ACTIN2* was used as an internal control. Data are means ± SD of three different experiments, with similar results.
(TIF)

S5 Fig Effects of SHAM and AsA on disease development in *Arabidopsis* treated with SA and infected with *Pst* DC3000. (**a**) The necrotic lesions on representative Arabidopsis leaves at 3 or 5 days after infected by *Pst* DC3000 in AsA- or SHAM-pretreated plants. 3-week-old Arabidopsis ecotype Col-0 plants were pretreated with AsA (1.5 mM) or SHAM (20 mM) before spraying with either water (Control) or SA, and then inoculated with *Pst* DC3000. Infection was observed 3 and 5 days after inoculation. (**b**) *Pst* DC3000 growth analysis in AsA- or SHAM-pretreated detached leaves of wild-type. FW, fresh weight. Asterisks indicate significant differences to control (student’s t-test: *p < 0.05), and #, P < 0.05 vs SA. Data are means ± SD of four different experiments, with similar results.
(TIF)

S6 Fig Analysis of effect of CsA on mitochondrial MTP in response to SA using fluorescence probe Rh-123. Protoplasts incubated in W5 medium containing 500 μM SA, 500 μM SA plus 5 μM CsA for the indicated time, and then the fluorescence intensity of Rh-123 was analyzed.
(TIF)

S1 Table Primers for *AOX1a* gene in real-time qPCR. (DOC)
