# Supplementary figures and images for: Mitochondrial-Derived Reactive Oxygen Species Play a Vital Role in the Salicylic Acid Signaling Pathway in Arabidopsis thaliana
Source: PLoS One. 2015 Mar 26;10(3):e0119853. doi: 10.1371/journal.pone.0119853 (PMC4374720; doi:10.1371/journal.pone.0119853)

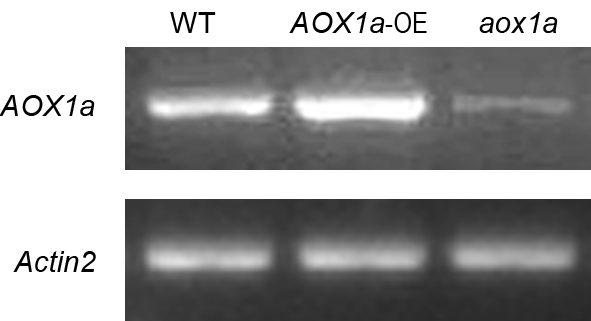

Supplement: S1 Fig — The total RNAs were extracted from the leaves of 2-week-old Arabidopsis wild-type, aox1a mutant and AOX1a-OE seedlings and analyze the transcripts by the semi-quantitative RT-PCR, and the Actin2 was analyzed as standard. (TIF) [file pone.0119853.s001.tif]

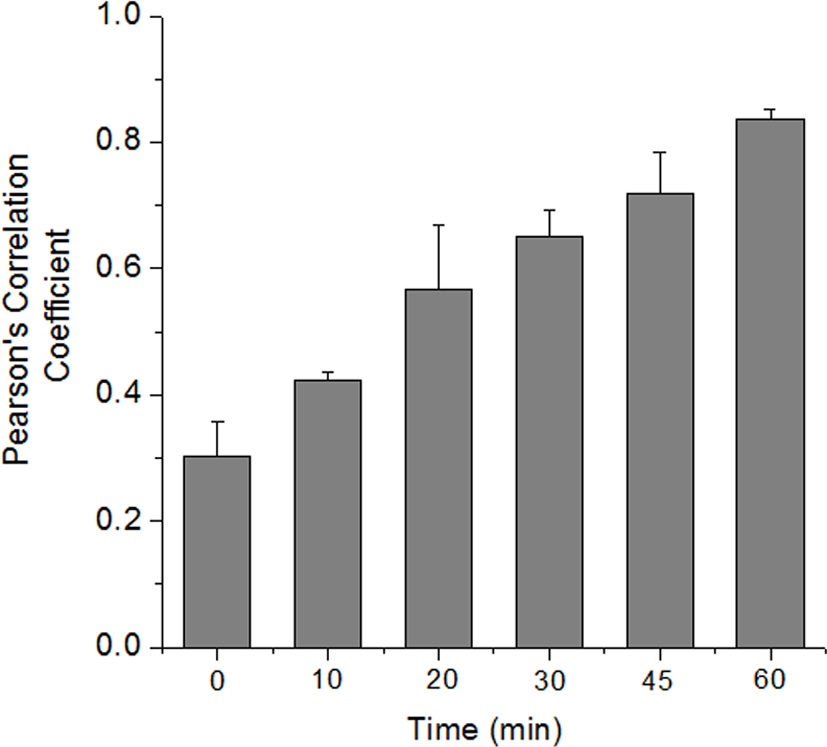

Supplement: S2 Fig — Protoplasts were treated with or without SA for the indicated time, double-stained with H2DCFDA and MitoTracker Red CMXRos (MT), and detected using a LCSM. The co-localization between DCF and MitoTracker Red CMXRos fluorescence was examined using IPP software. Data are means ± SD of three different experiments, with similar results. (TIF) [file pone.0119853.s002.tif]

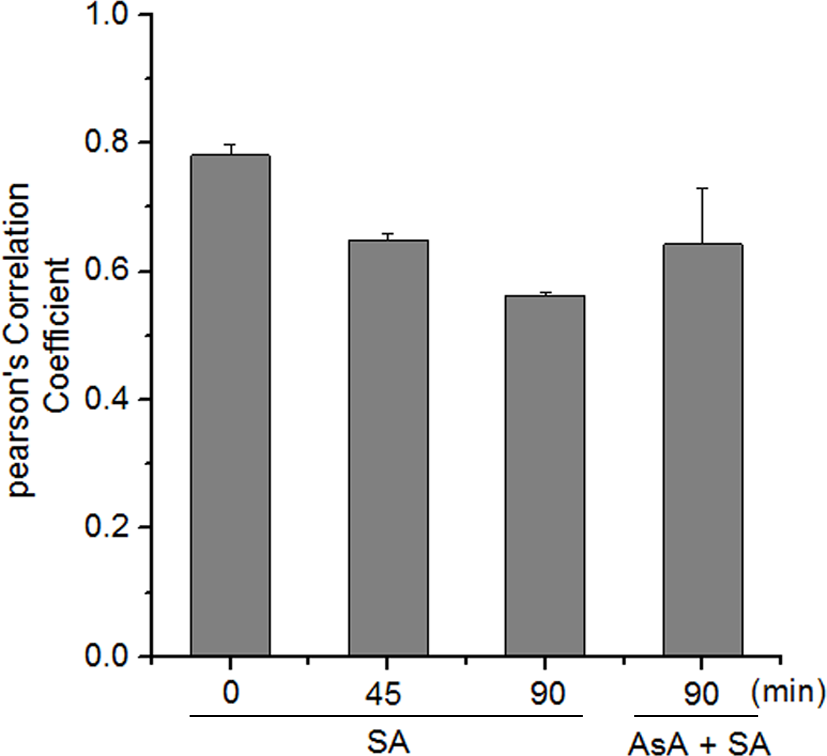

Supplement: S3 Fig — Protoplasts were pre-incubated with or without AsA at 1 mM final concentration for 30 min, and were then untreated for 1.5 h or treated with SA for 1.5 h. Samples were double stained with Rh-123 and MitoTracker Red CMXRos and observed with a LCSM. The co-localization between Rh123 and MitoTracker Red CMXRos fluorescence was examined using IPP software. Data are means ± SD of three different experiments, with similar results. (TIF) [file pone.0119853.s003.tif]

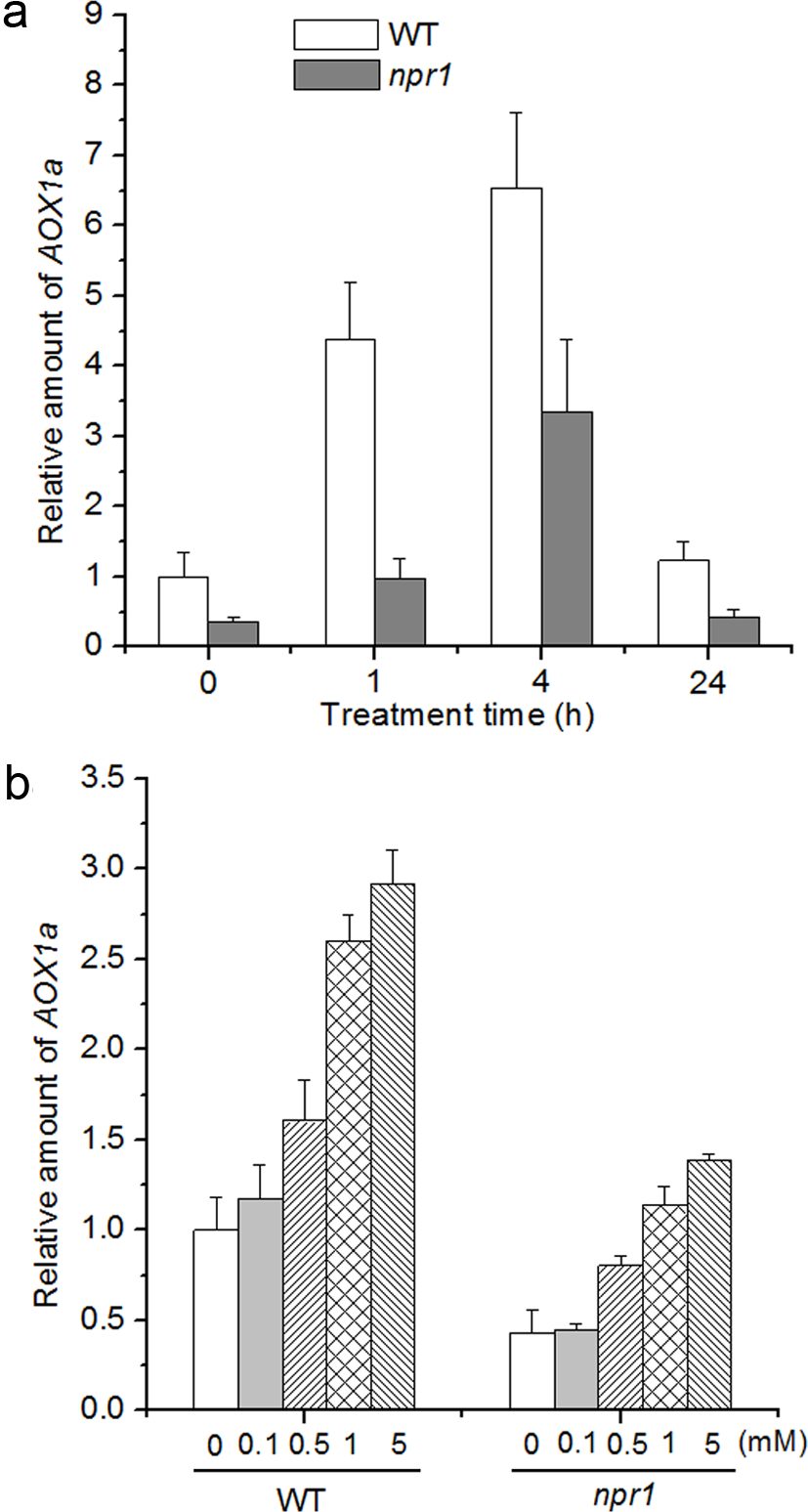

Supplement: S4 Fig — (a) Real-time quantitative RT-PCR of Aox1a in WT and npr1 mutant plants from control (0 h) and seedlings treated with 500 μM SA for 1, 4 and 24 h. Statistical analysis was performed with Student’s t-test: *, P < 0.05 vs 0 h. (b) Effect of H2O2 on AOX1a gene expression in WT and npr1 mutant. Seedlings were treated with increasing concentrations of H2O2 (0–5 mM), and the expression of AOX1a was analyzed by real-time PCR. Arabidopsis ACTIN2 was used as an internal control. Data are means ± SD of three different experiments, with similar results. (TIF) [file pone.0119853.s004.tif]

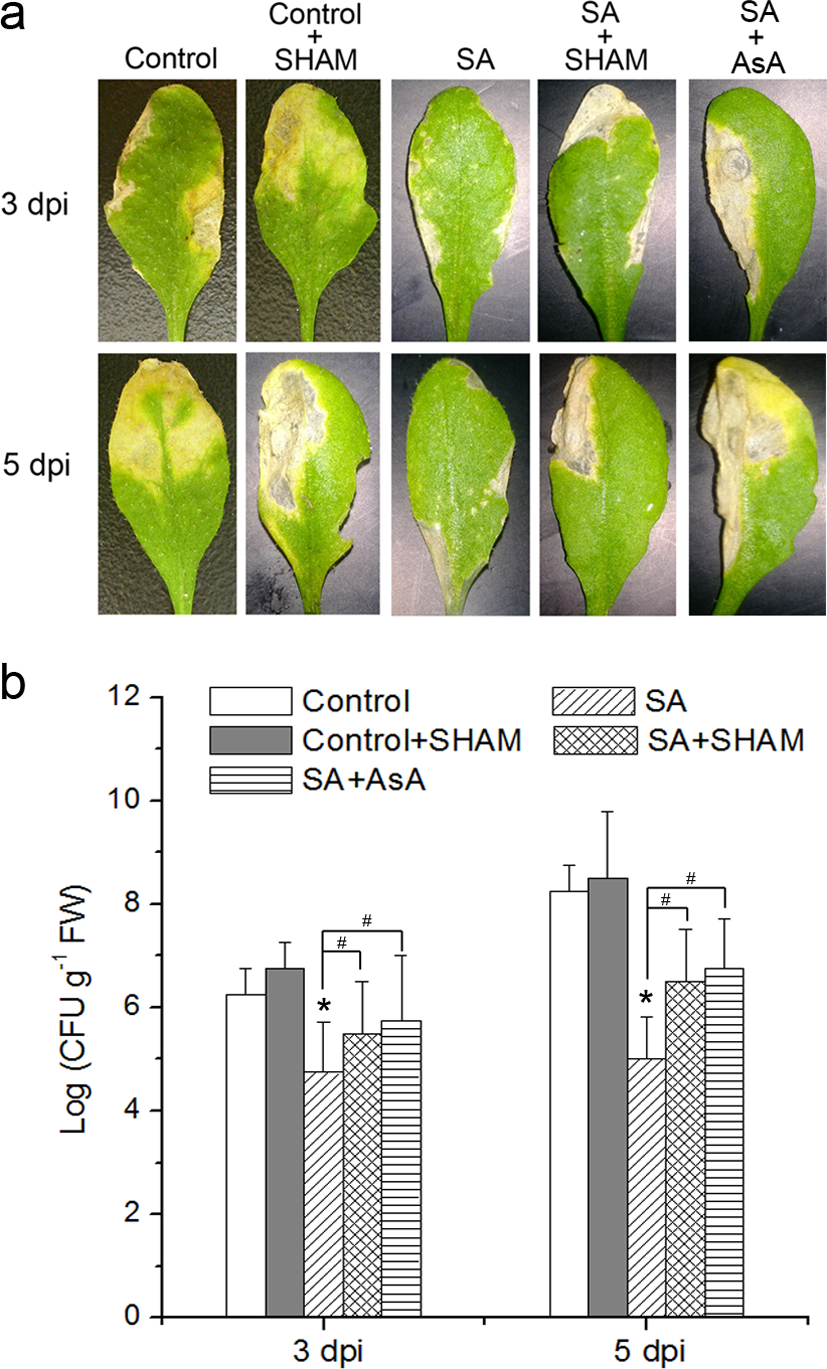

Supplement: S5 Fig — (a) The necrotic lesions on representative Arabidopsis leaves at 3 or 5 days after infected by Pst DC3000 in AsA- or SHAM-pretreated plants. 3-week-old Arabidopsis ecotype Col-0 plants were pretreated with AsA (1.5 mM) or SHAM (20 mM) before spraying with either water (Control) or SA, and then inoculated with Pst DC3000. Infection was observed 3 and 5 days after inoculation. (b) Pst DC3000 growth analysis in AsA- or SHAM-pretreated detached leaves of wild-type. FW, fresh weight. Asterisks indicate significant differences to control (student’s t-test: *p < 0.05), and #, P < 0.05 vs SA. Data are means ± SD of four different experiments, with similar results. (TIF) [file pone.0119853.s005.tif]

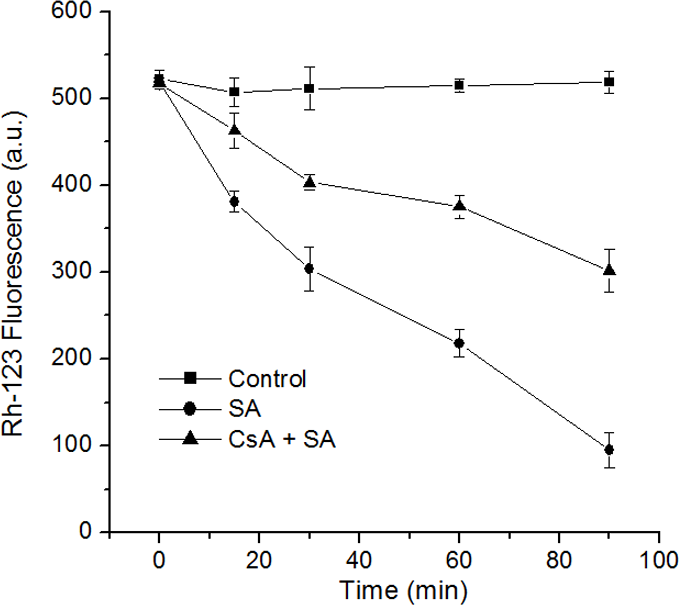

Supplement: S6 Fig — Protoplasts incubated in W5 medium containing 500 μM SA, 500 μM SA plus 5 μM CsA for the indicated time, and then the fluorescence intensity of Rh-123 was analyzed. (TIF) [file pone.0119853.s006.tif]
