# Supplementary material for: Mitochondrial-Derived Reactive Oxygen Species Play a Vital Role in the Salicylic Acid Signaling Pathway in Arabidopsis thaliana
Source: PLoS One. 2015 Mar 26;10(3):e0119853. doi: 10.1371/journal.pone.0119853 (PMC4374720; doi:10.1371/journal.pone.0119853)
Supplement: S1 Table — (DOC) [file pone.0119853.s007.doc]

**Mitochondrial-derived reactive oxygen species play a vital role in the salicylic acid signaling pathway in *Arabidopsis thaliana***

Shengjun Nie, Haiyun Yue, Jun Zhou and Da Xing*

**S1 Table. Primers for AOX1a gene in qPCR.**

| Gene Name | Primer pairs (5’-3’) |
| --- | --- |
| *AOX1a* | F: ATGATGATAACTCGCGGTGGAGC |
|  | R: GCAACATTCAAAGAAAGCCGAATC |
| *ACTIN2* | F: AGAGATTCAGATGCCCAGAAGTCTTGTTCC |
|  | R: AACGATTCCTGGACCTGCCTCATCATACTC |

Note: F indicates forward and R indicates reverse.
